# Effectiveness comparisons of drug therapies for postoperative aneurysmal subarachnoid hemorrhage patients: network meta‑analysis and systematic review

**DOI:** 10.1186/s12883-021-02303-8

**Published:** 2021-07-27

**Authors:** Wanli Yu, Yizhou Huang, Xiaolin Zhang, Huirong Luo, Weifu Chen, Yongxiang Jiang, Yuan Cheng

**Affiliations:** 1 Department of Neurosurgery, The Second Affiliated Hospital, Chongqing Medical University, Chongqing, China; 2 Department of Endocrinology, The Second Affiliated Hospital, Chongqing Medical University, Chongqing, China; 3 Department of Psychiatry, The First Affiliated Hospital, Chongqing Medical University, Chongqing, China

**Keywords:** Aneurysmal subarachnoid hemorrhage, Clinical outcome, Drug therapies, Network meta-analysis

## Abstract

**Objective:**

To compare the effectiveness of various drug interventions in improving the clinical outcome of postoperative patients after aneurysmal subarachnoid hemorrhage (aSAH) and assist in determining the drugs of definite curative effect in improving clinical prognosis.

**Methods:**

Eligible Randomized Controlled Trials (RCTs) were searched in databases of PubMed, EMBASE, and Cochrane Library (inception to Sep 2020). Glasgow Outcome Scale (GOS) score, Extended Glasgow Outcome Scale (GOSE) score or modified Rankin Scale (mRS) score was used as the main outcome measurements to evaluate the efficacy of various drugs in improving the clinical outcomes of postoperative patients with aSAH. The network meta-analysis (NMA) was conducted based on a random-effects model, dichotomous variables were determined by using odds ratio (OR) with 95% confidence interval (CI), and a surface under the cumulative ranking curve (SUCRA) was generated to estimate the ranking probability of comparative effectiveness among different drug therapies.

**Results:**

From the 493 of initial citation screening, forty-four RCTs (n = 10,626 participants) were eventually included in our analysis. Our NMA results showed that cilostazol (OR = 3.35,95%CI = 1.50,7.51) was the best intervention to improve the clinical outcome of patients (SUCRA = 87.29%, 95%CrI 0.07–0.46). Compared with the placebo group, only two drug interventions [nimodipine (OR = 1.61, 95%CI 1.01,2.57) and cilostazol (OR = 3.35, 95%CI 1.50, 7.51)] achieved significant statistical significance in improving the clinical outcome of patients.

**Conclusions:**

Both nimodipine and cilostazol have exact curative effect to improve the outcome of postoperative patients with aSAH, and cilostazol may be the best drug to improve the outcome of patients after aSAH operation. Our study provides implications for future studies that, the combination of two or more drugs with relative safety and potential benefits (e.g., nimodipine and cilostazol) may improve the clinical outcome of patients more effectively.

**Supplementary Information:**

The online version contains supplementary material available at 10.1186/s12883-021-02303-8.

## Introduction

Subarachnoid hemorrhage (SAH) can occur at any age, especially at 40–60 years old [1]. It has been reported that the global morbidity of SAH is about 6.1 per 100,000 person-years [2] and 85% of patients are caused by ruptured intracranial aneurysms [3]. As an extremely devastating disease, spontaneous aneurysmal Subarachnoid Hemorrhage (aSAH) has a case-fatality rate of 10.9%-27.5% [4–7], 22–28.7% [7, 8] and 30.7% [8] during hospitalization, at 30 days and at three months, respectively. Moreover, at least 10%-15% of aSAH patients have died before arriving at the hospital, missing the chance of proper rescue [9]. Early surgical interventions (Craniotomy Clipping and Endovascular Embolization) of ruptured aneurysms can effectively reduce the incidence of rebleeding and mortality of patients. However, even for patients who survive after operation, there remains high risks of early mortality and long-term disability [10]. Several literature illustrated that the early mortality rate of aSAH has decreased [7, 11–13] by approximately 25–30% [8, 14], but more than 50% of the postoperative survivors still progress into a state of severe disabilities [15]. Under the condition with well controlled risk of rebleeding, comprehensive postoperative management should be highlighted to improve the overall outcome of patients, which indicates the necessity for optimal application of drug therapies. During the past few decades, various drugs were applied to postoperative aSAH whereas there was still an absence of comprehensive comparisons between those drugs. Based on the above problems, we had analyzed the existing evidence to summarize the best drug which could improve the clinical outcome of postoperative patients with aSAH. In addition, a hierarchical ordering was performed to help clinicians make individual decisions based on the effectiveness of multiple drugs.

## Methods

This review followed guidance for the conduct and reporting of systematic reviews from the Cochrane handbook [16] and the PRISMA NMA checklist [17]. The protocol of this review was registered on PROSPERO (ID: CRD 42,020,219,424).

### Search strategy and study selection

The search strategy was designed and implemented separately by two authors. A comprehensive search of all the randomized controlled trials (RCTs) of aSAH drug treatment was conducted at PubMed, Cochrane Library, Embase from their inception to September 10, 2020. We rigorously compare the effects of two or more treatments groups or between the treatment group and the control group (placebo, inactive group) in the treatment of aSAH. Without being restricted by year and language, the Medical Subject Headings (MeSH) combined with free words followed by Boolean logical operators were conducted by using “aSAH”, “Nimodipine”, “Nicardipine”, “Magnesium”, “Milrinone”, “Statins”, “Clazosentan”, “Tirilazad”, “Fasudil”, “Cilostazol”, “methylprednisolone”, “Enoxaparin”, “Randomized controlled trials” as well as additional relevant conceptual keywords. All analyses were based on previously published research and did not require ethical approval or patient consent.

According to predefined selection criteria, two authors independently evaluated all available citations. We screened the titles and abstracts of articles obtained from the search first, and excluded articles which did not meet the inclusion criteria or were repeatedly published. For research published many times, we chose the most informative and complete manuscript. For articles that may meet the inclusion criteria, two authors (WLY, YZH) carefully read the full text to further evaluate their relevance. In addition, the references included in those articles were also evaluated to further explore the relevant research. All the citations were downloaded and managed in accordance with the prespecified standards in Endnote X9 (Thompson ISI Research Soft, Philadelphia, PA, USA). In order to ensure smooth proceeding of further analysis, it is necessary to check the accuracy and completeness of the data. Any discrepancies in search strategy and article inclusion process were resolved through discussion or arbitration by two experienced authors (YC, YXJ).

### Inclusion & exclusion criteria and outcome measurement

Inclusion criteria: (1) The included patients need to be diagnosed as aSAH through clear imaging characteristics and clinical manifestations, and all of patients need to be treated with coiling or clamping within 72 h after hospitalisation; (2) Clearly reported outcome indicators; (3) At least ten aSAH patients; (4) published in English between 1980 and 2019; (5) Peer-reviewed original RCTs;

Exclusion criteria: researches that applied two or more drug interventions simultaneously;

According to the inclusion and exclusion criteria, two authors (WLY, YZH) used The Cochrane Consumers and communication Review Group’s data extraction template [18] to extract and organize data for qualified studies independently. We first analyzed the global data and demographic characteristics of all included studies according to the pre-customized outcome data collection form. The following relevant data were extracted by two authors (WLY, HRL) as baseline data including: name of studies, first author of article, year of publication, country and region, duration of treatment, and basic characteristics. Placebo was the designated control group (DCG) for pair-wise and network meta-analyses.

Glasgow Outcome Scale (GOS) score, Extended Glasgow Outcome Scale (GOSE) score or modified Rankin Scale (mRS) score was used as the main outcome measurements to evaluate the efficacy of various drugs in improving the clinical outcomes of postoperative patients with aSAH. Good outcome was defined as no disability or moderate disability, including GOS > 3 or mRS < 4 or GOSE > 4. Poor outcome included severe disability, vegetative state and death (GOS ≤ 3 or mRS ≥ 4 or GOSE ≤ 4). Each clinical study was followed up for at least 2 weeks.

### Data abstraction and quality appraisal

In the process of extracting data, any disagreements were resolved through discussion between pairs of authors. Experienced professor (YC) was invited to judge the disagreements objectively if necessary. Data could be then entered with accuracy and unanimity.

We used Cochrane Risk of Bias tool to assess the risk of bias (ROB) of included studies [16]. Seven domains of ROB were evaluated by two authors separately to define each study as of high, low, or unclear risk of bias, including random sequence generation, allocation concealment, blinding of participants and personnel, blinding of outcome assessment, incomplete outcome data, selective reporting, and other bias. The evaluation of ROB was carried out in software of Review Manager (Version 5.4).

### Statistical analyses

The Frequentist and Bayesian network meta-analysis has stronger classification capabilities than traditional meta-analysis because it can summarize the comparisons between multiple therapies at the same time, making complex models more flexible, and generating relatively scientific interpretation in terms of causal relationships [19]. We used minimally informative prior distributions based on random effect statistical model to integrate direct and indirect evidence and compare various drug interventions by forming a connected network. We first performed a traditional pairwise meta-analysis for each control. In terms of statistical heterogeneity, 25%, 50%, 75% *I*^*2*^ statistic was used to evaluate the heterogeneity of each comparative test [20]. a random-effect based comparison-adjusted funnel plot was conducted to detect the presence of any dominant types of bias, such as publication bias, selective reporting or other biases.

We draw a network plot as a simple summary description to present all the available evidence of each treatment evidence and sequence of analyses were performed in STATA, version 16.0 (Corporation LLC, College Station, USA). We set the odds ratio (OR) as a 95% Confidence Interval (CI) for the dichotomous result.

In order to ensure that different treatment comparisons were sufficiently similar to provide valid indirect inferences, we achieved the transitivity assumption by comparing all the clinical and methodological characteristics of the included studies, such as patients and experimental designs. The hierarchical random effects were used to compare multiple drug interventions at the same time by forming a connected network, integrating direct and indirect estimates and using the methodology of multivariate meta-analysis. In the case of randomly selecting state, three parallel Markov chains were initially established to simulate the statistical models for accurate evaluate [21]. Each chain generated 50,000 iterations, and the first 10,000 iterations were discarded to ensure that the bias of initial values were minimized when the chain reached its target distribution. The Brooks-Gelman-Rubin diagnosis method was used to assess the convergence of models by examining the history trajectory of trace plot combined with density plot [22]. We use OpenBUGS (version 3.2.3 rev 1012) calculated treatment rank probability and the surface under the cumulative ranking curve (SUCRA) was generated to display a simple numerical statistical cumulative ranking probability plots of various interventions. SUCRA would be 1 if a treatment is certainly at the highest level or highly effective, while zero undoubtedly means that the treatment is the worst [23]. What’s more, we used the "node-splitting" technique [24] to compare the indirect evidence from the entire network with direct evidence in order to explore whether there will be a potential source inconsistence in our network. (*p* value > 0.05 indicates the consistency) [25].

## Results

### Baseline characteristics and ROB quality assessment

**Fig. 1:**
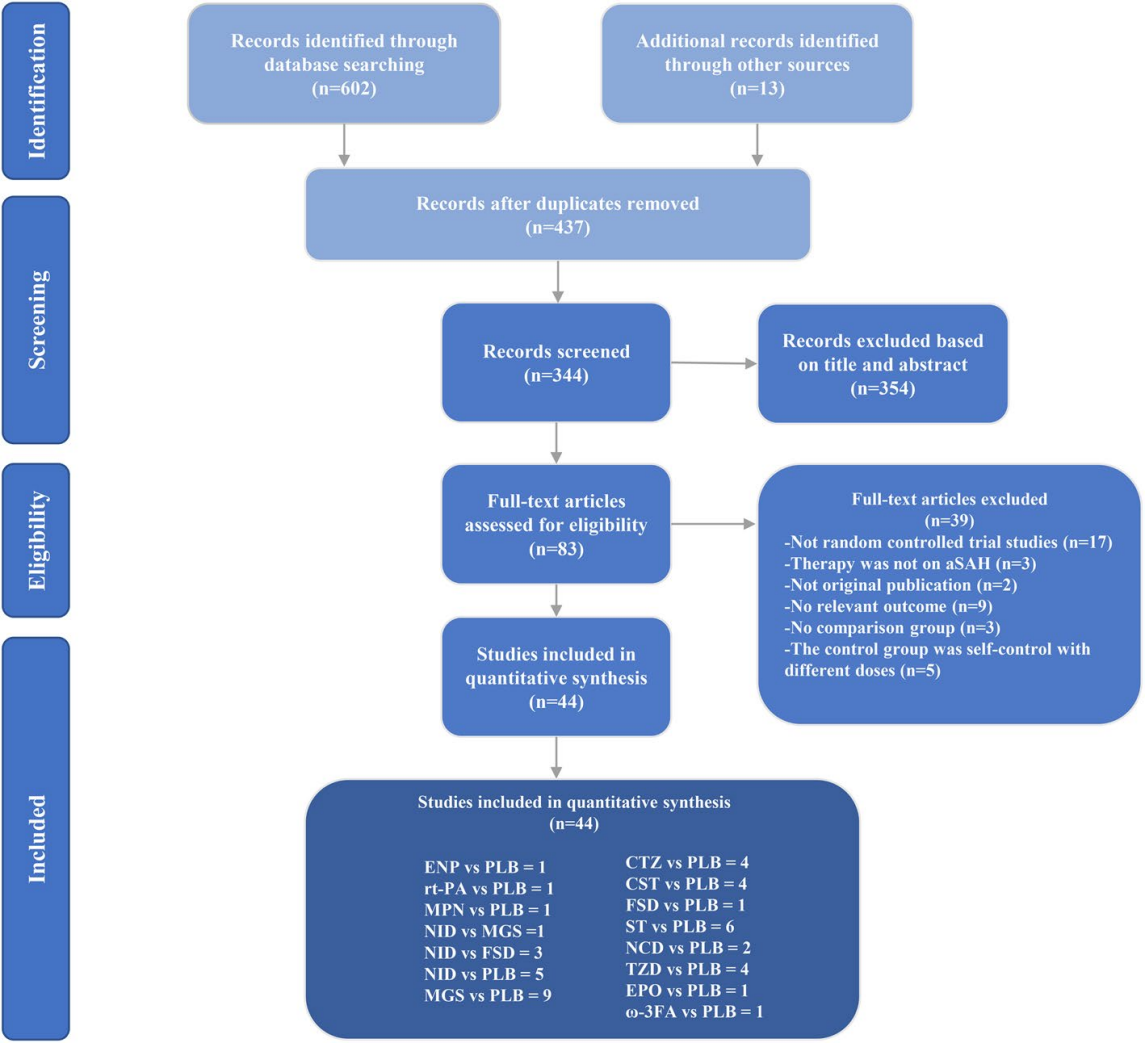
legend Literature screening flowchart. *NID* nimodipine, *MGS* magnesium, *CTZ* cilostazol, *CST* clazosentan, *FSD* fasudil, *NCD* nicardipine, *TZD* tirilazad, *ENP* Enoxaparin, *EPO* erythropoietin, *M*PN methylprednisolone, ST statins, rt-PA Recombinant Human Tissue-type plasminogen activator, *ω-3FA* Omega-3 fatty acid

602 articles were initially screened through searching of databases and 13 additional articles were obtained by tracking the references from initially screened articles. Then we eliminated 165 of duplicates and other 354 articles after reading the title and abstract. Based on the full-text examination, 39 articles were excluded as 17 articles were not RCTs, 3 articles were not about treatment for aSAH or was animal experiment, 2 articles were not original research, 9 articles without relevant outcome or reported data can’t be extracted, 3 articles without a control group, and 5 articles were self-controls with different doses in the control group. Finally, 44 articles, including 13 drug interventions, and a total of 10,626 patients were included in this NMA. The processing of literature selection is shown in Fig. 1.

The included studies provided data published from 1986 to 2017. Table 1 summarizes the key characteristics of participants and interventions of the 44 included trials. All cases included in each study were patients with aSAH. The severity of the disease varied [Hunt-Hess grade 1–5 or World Federation of Neurological Surgeons (WFNS) grade 1–5], and each clinical study was followed up for at least 2 weeks. The duration of studies varied from 3 to 76 days. According to available data, 66.28% of patients were women. 17 RCTs consisted of 4,527 participants from Europe, followed by Asia which contained 16 RCTs with 3392 participants, 6 RCTs were comprised of participants from multiple continents, and the remaining 5 RCTs were originated from the USA and Africa.

**Table 1:** Characteristics of included studies

Publication	Treatments and sample size	Endpoints	Mean age (years, ± SD)	Proportion of girls	Treatment duration	Recruiting area
Petruk, 1988 [26]	NID = 72versus PLB = 82	GOS,3 months	47.1 ± 1.0	66.9%	21d	UK
Schmid-elsaesser, 2006 [27]	NID = 51versus PLB = 53	GOS,12 months	53.0 ± 16.0	58.7%	21d	Germany
Pickard, 1989 [28]	NID = 278versus PLB = 276	GOS,3 months	47.0 ± 1.0	60.1%	21d	UK
Jan, 1988 [29]	NID = 73versus PLB = 54	GOS,14 days	48.0 ± 0.6	53.5%	7-14d	France
Ohman, 1991 [30]	NID = 104versus PLB = 109	GOS,21 days	45.2 ± 11.2	51.4%	21d	Finland
Zhao, 2011 [31]	NID = 60versus FSD = 55	GOS,1 month	50.0 ± 11.4	61.2%	14d	China
Philippon, 1986 [32]	NID = 31versus PLB = 39	GOS,21 days	45.0 ± 12.9	57.1%	21d	France
Westermaier, 2010 [33]	MGS = 54versus PLB = 53	GOS,6 months	51.0 ± 13.0	38.3%	10d	Germany
Boet, 2005 [34]	MGS = 23versus PLB = 22	GOS,3 months	57.0	45.1%	14d	Hong Kong, China
Muroi, 2008 [35]	MGS = 27versus PLB = 31	GOS,3 months	52.8 ± 12.7	25.9%	12d	Switzerland
Vandenbergh, 2005 [36]	MGS = 122versus PLB = 127	mRS,3 months	54.5 ± 0.1	67.1%	14d	Netherlands
Akdemir, 2009 [37]	MGS = 40versus P = 43	GOS,3 months	53.7 ± 0.3	61.4%	10d	Turkey
Mees, 2012 [38]	MGS = 606versus PLB = 597	mRS,3 months	57.0	69.7%	20d	Netherlands/Chile/UK
Wong, 2006 [39]	MGS = 30versus PLB = 30	GOS,6 months	60.0 ± 2.0	70.0%	14d	Hong Kong, China
Wong, 2010 [40]	MGS = 169versus PLB = 158	mRS,6 months	57.0	63.6%	14d	Hong Kong, China
Hassan, 2011 [41]	MGS = 15versus PLB = 15	GOS,3 months	49.5 ± 0.5	70.0%	14d	Egypt
Matsuda, 2016 [42]	CTZ = 74versus PLB = 74	GOS,3 months	58.5 ± 12.0	67.6%	14d	Japan
Senbokuya, 2013 [43]	CTZ = 54versus PLB = 55	GOS,3 months	60.7 ± 12.6	62.4%	14d	Japan
Suzuki, 2011 [44]	CTZ = 49versus PLB = 51	mRS,14 days	63.0 ± 13.5	76.0%	14d	Japan
Yoshimoto, 2009 [45]	CTZ = 26versus PLB = 24	mRS,1 month	59.0 ± 1.0	74.0%	14d	Japan
Macdonald, 2008 [46]	CST = 107versus PLB = 96	GOSE,3 months	51 ± 10.5	73.9%	14d	11 countries in Europe
Macdonald, 2011 [47]	CST = 764versus PLB = 383	GOSE,3 months	51.7 ± 11.0	67.6%	14d	27 countries worldwide
Macdonald, 2012 [48]	CST = 181versus PLB = 172	GOSE,3 months	53.0 ± 1.0	70.2%	14d	27 countries worldwide
Fujimura, 2017 [49]	CST = 52versus PLB = 55	GOSE,3 months	55.2 ± 11.2	60.0%	14d	Japan/Korea
Shibuya, 1992 [50]	FSD = 131versus PLB = 136	GOS,1 month	55.0 ± 11.0	56.2%	14d	Japan
Zhao, 2006 [51]	FSD = 33versus NID = 34	GOS,1 month	50.1 ± 11.4	61.1%	14d	China
JingjianMA, 2009 [52]	FSD = 32versus NID = 32	GOS,14 days	48.5 ± 10.0	62.5%	14d	China
Tseng, 2005 [53]	ST = 40versus PLB = 40	mRS,14 days	52.9 ± 12.0	55.0%	14d	UK
Garg, 2013 [54]	ST = 19versus PLB = 19	GOS,3 months	49.1 ± 1.6	55.3%	14d	India
Naraoka, 2017 [55]	ST = 54versus PLB = 54	GOS,3 months	56.5 ± 1.5	68.5%	14d	Japan
Vergouwen, 2009 [56]	ST = 1 6versus PLB = 16	GOS,3 months	53.5 ± 0.5	62.5%	14d	Netherlands
Chou, 2008 [57]	ST = 19versus PLB = 20	mRS,21 days	53.1 ± 14.6	74.4%	21d	USA
Kirkpatrick, 2014 [58]	ST = 379versus PLB = 403	mRS,6 months	50.0 ± 9.7	62.7%	21d	UK/Other countries
Haley, 1993 [59]	NCD = 447versus PLB = 455	GOS,3 months	49.9 ± 14.0	63.8%	14d	USA/ Canada
Barth, 2006 [60]	NCD = 12versus PLB = 12	mRS,12 months	52.5 ± 7.1	27.0%	14d	Germany
Haley, 1995 [61]	TZD = 61versus PLB = 42	GOS,3 months	50.2 ± 14.0	61.2%	21d	Canada
Kassell, 1996 [62]	TZD = 251versus PLB = 256	GOS,3 months	50.1 ± 13.4	64.5%	10d	Europe/Australia/New Zealand
Haley, 1997 [63]	TZD = 300versus PLB = 299	GOS,3 months	51.0 ± 13.1	68.5%	10d	USA
Lanzino, 1999 [64]	TZD = 405versus PLB = 414	GOS,3 months	53.0	100.0%	10d	Europe/Australia/New Zealand/South Africa
Springborg, 2007 [65]	EPO = 24versus PLB = 30	GOS,6 months	54.6 ± 11.3	72.2%	3d	Denmark
Nakagawa, 2016 [66]	ω-3FA = 55versus PLB = 45	mRS,3 months	62.2 ± 2	52.4%	76d	Japan
Siironen, 2003 [67]	HPN = 85versus PLB = 85	GOS,3 months	49.9 ± 1.4	51.2%	10d	Finland
Etminan, 2013 [68]	rt-PA = 30versus PLB = 30	GOS,3 months	56.1 ± 10.4	63.3%	11d	Germany
Gomis, 2010 [69]	MPN = 49versus PLB = 46	GOS,12 months	49.8 ± 13.2	63.2%	21d	France

*NID* nimodipine, *MGS* magnesium, *CTZ* cilostazol, *CST* clazosentan, *FSD* fasudil, *NCD* nicardipine, *TZD* tirilazad, *HPN* heparin, *ENP* Enoxaparin, *EPO* erythropoietin, *MPN* methylprednisolone, *ST* statins, *GOS* Glasgow Outcome Scale, *mRS* Modified Rankin Scale, *GOSE* Glasgow Outcome Scale Extended, *PLB* placebo

Individual and overall study-level quality were summarized in Supplement Fig. 1 and Supplement Fig. 2, respectively. Within the 44 included trials, 41 trials described in detail the generation of random sequences, 38 trials described their approach of concealment, 26 studies did not describe the blind methods of participants, implementers, or outcome measurers. 38 studies have complete data, and only 5 studies may have reporting bias.

**Fig. 2:**
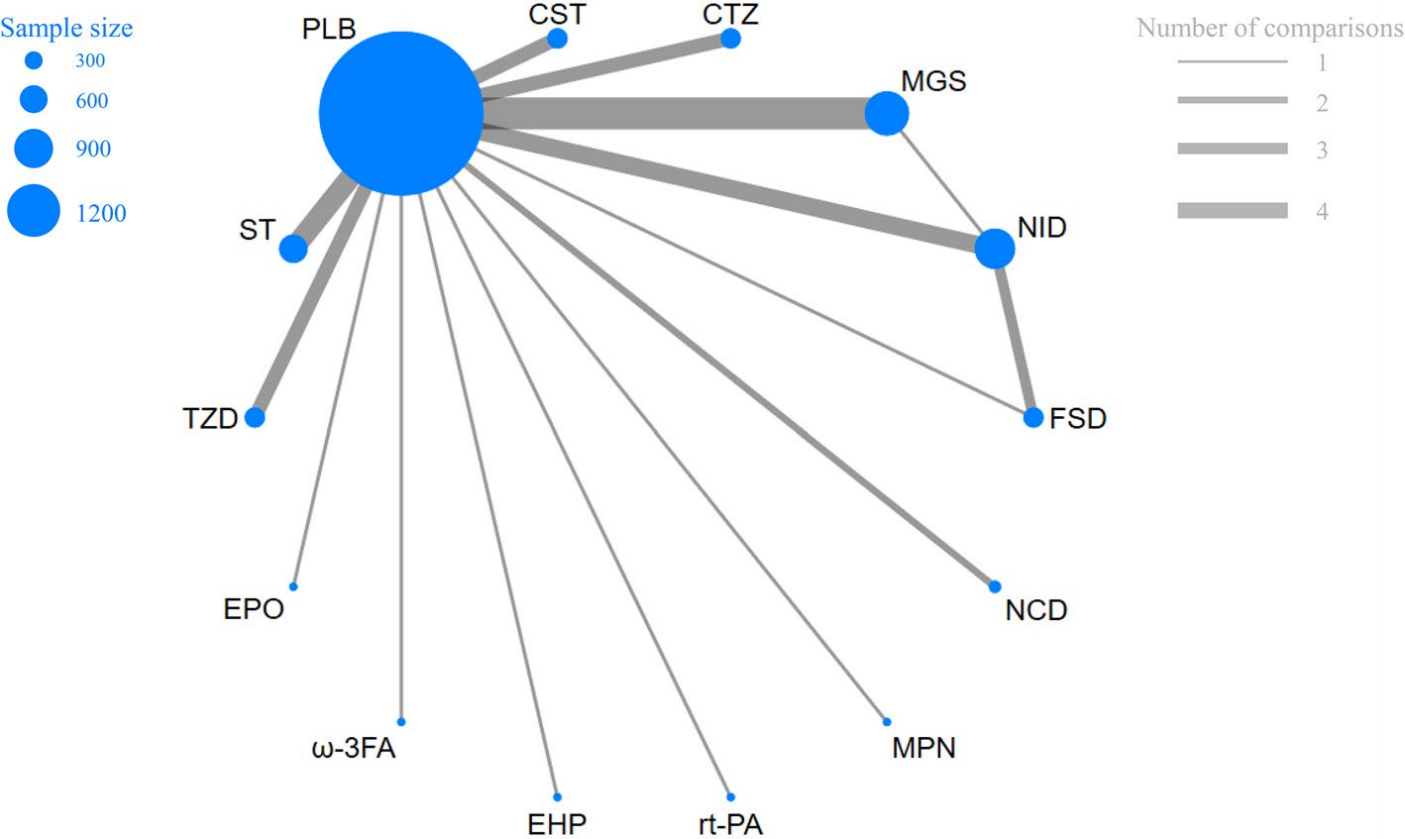
legend Network of evidence of the included trials

### Pairwise meta‑analysis and NMA results

The funnel plot illustrated that publication bias generation relies on the distribution of some scattering spots which are asymmetrical in the inverted funnel plot (Supplementary Fig. 3).

As shown in Fig. 2, the network geometry was visualized to display each arm. Each treatment has its own unique node, whose size depends on their contribution to the entire network. In our NMA, comparisons between thirteen drug intervention groups were described. Magnesium (MGS) was most frequently included with 10 arms (n = 1139), followed by nimodipine (NID) involving 9 arms (n = 735), statins (ST) involving 6 arms (n = 527), cilostazol (CTZ) involving 4 arms (n = 203), clazosentan (CST) involving 4 arms (n = 276), tirilazad (TZD) involving 4 arms (n = 1017), fasudil (FSD) involving 3 arms (n = 196), nicardipine (NCD) involving 2 arms (n = 459), and erythropoietin (EPO) involving 1 arm (n = 24), Omega-3 fatty acid (ω-3FA) involving 1 arm (n = 55), enoxaparin (ENP) involving 1 arm (n = 85), Recombinant Human Tissue-type plasminogen activator (rt-PA) involving 1 arm (n = 30), methylprednisolone (MPN) involving 1 arm (n = 49), among which 4 studies were direct trials.

**Fig. 3:**
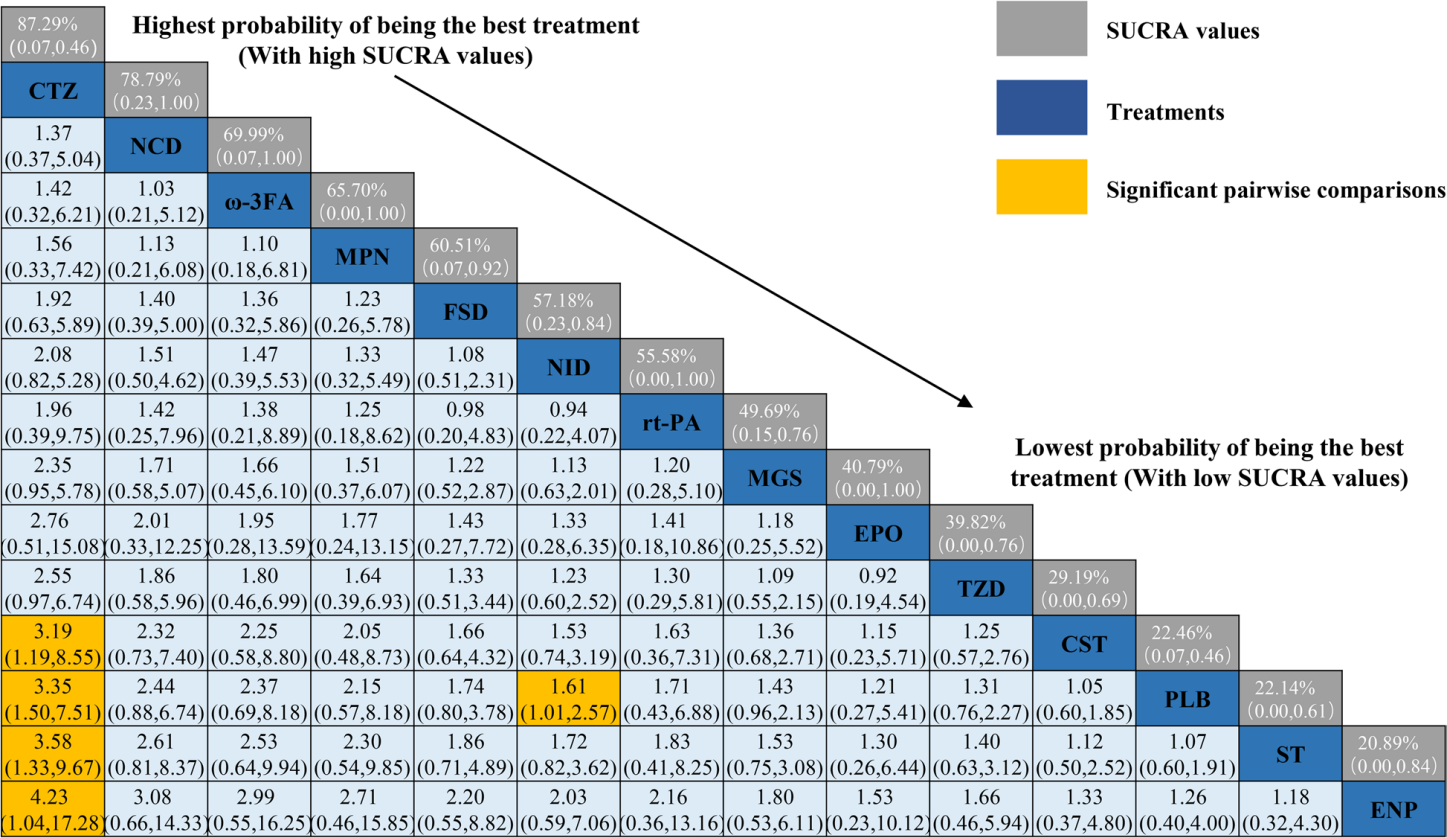
legend Relative effect sizes of efficacy at post-treatment according to network meta-analysis. Treatments are ranked according to their chance of being the best treatment. Numbers in the gray boxes are the values of SUCRA (the surface under the cumulative ranking curve), which represents the rank of treatment. Significant pairwise comparisons are highlighted in orange. In terms of post-treatment efficacy, patients with OR (odds ratio) less than 1 favor the designated control group (DCG)

As shown in Fig. 3, a total of 2 drugs were statistically significant superior to placebo group, including NID (OR = 1.61, 95%CI 1.01,2.57) and CTZ (OR = 3.35, 95%CI 1.50, 7.51). In addition, the efficacy of CTZ was significantly higher than CST, ST and HPN [CST (OR = 3.19, 95%Cl 1.19,8.55), ST (OR = 3.58, 95%Cl 1.33,9.67), HPN (OR = 4.23, 95%Cl 1.04,17.28), respectively]. The remaining NCD (OR = 2.44, 95%Cl 1.50,7.51), ω-3FA (OR = 2.37, 95%Cl 0.69,8.18), MPN (OR = 2.15, 95%Cl 0.57,8.18), FSD (OR = 1.74, 95%Cl 0.80,3.78), rt-PA (OR = 1.71, 95%Cl 0.43,6.88), MGS (OR = 1.43, 95%Cl 0.96,2.13), TZD (OR = 1.31, 95%Cl 0.76,2.27), EPO (OR = 1.21, 95%Cl 0.27,5.41), CST (OR = 1.05, 95%Cl 0.60,1.85) were more likely to have a good prognosis than the placebo group (GOS > 3 or mRS < 4). The efficacy of ST (OR = 0.94, 95%Cl 0.52,1.67) and ENP (OR = 0.79, 95%Cl = 0.25,2.51) may not be as good as the placebo group, but the differences were not statistically significant.

We plotted the SUCRA line to rank each drug intervention (shown in Supplement SUCRA plot and Table 2), which illustrated that compared with other 12 drug interventions, CTZ had the highest probability of improving the prognosis of aSAH patients (SUCRA = 87.29%, 95%CrI 0.07–0.46) while NCD (SUCRA = 78.79%, 95%CrI 0.23–1.00) and ω-3FA (SUCRA = 69.99%, 95%CrI 0.07–1.00) also had a good ranking among the 13 interventions. The remaining MPN (SUCRA = 65.70%, 95%CrI 0.00–1.00), FSD (SUCRA = 60.51%, 95%CrI 0.07–0.92), NID (SUCRA = 57.18%, 95%CrI 0.23–0.84), rt-PA (SUCRA = 55.58%, 95%CrI 0.00–1.00), MGS (SUCRA = 49.69%, 95%CrI 0.15–0.76), EPO (SUCRA = 40.79%, 95%CrI 0.00–1.00), TZD (SUCRA = 39.82%, 95%CrI 0.00–0.76), CST (SUCRA = 29.19%, 95%CrI 0.00–0.69), PLB (SUCRA = 22.46%, 95%CrI 0.07–0.46), ST (SUCRA = 22.14%, 95%CrI 0.00–0.61) and ENP (SUCRA = 20.89%, 95%CrI 0.00–0.84) had an inferior ranking. There is no statistically significant inconsistency between direct or indirect comparison detected by node-splitting approach (PLB vs. NID p value = 0.343, PLB vs. MGS p value = 0.638, PLB vs. FSD p value = 0.430, NID vs. MGS p value = 0.638, NID vs. FSD p value = 0.430).

**Table 2:**
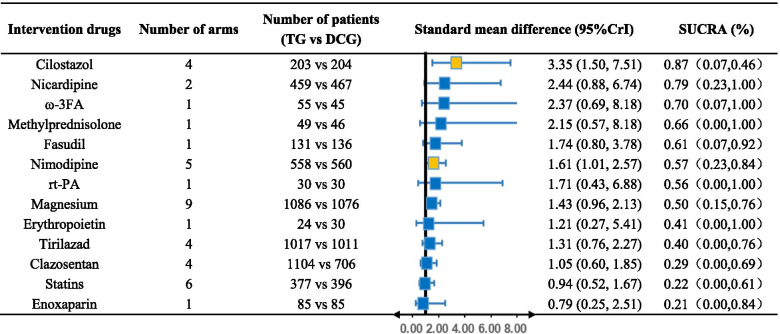
Efficacy of different intervention drugs compared to designated control group TG treatment group, DCG designated control group, 95%CrI 95% credibility interval, SUCRA the surface under the cumulative ranking curve

## Discussion

CTZ emerged as the best intervention to improve the clinical outcome of patients (SUCRA = 87.29%, 95%CrI 0.07–0.46) according to the NMA on postoperative drug treatments. Compared with the placebo group, only two drug interventions [NID (OR = 1.61, 95%CI 1.01,2.57), CTZ (OR = 3.35, 95%CI 1.50,7.51)] achieved significant statistics in improving the prognosis of patients.

Among our study, NID is the only drug approved by the FDA with neuroprotection and ability to improve the outcome of aSAH [70, 71]. NID has been considered to improve brain vasospasm for a long time, however, some early critical RCTs [26, 28–30] have shown seemingly contradictory results as there is a lack of correlation between the improvement of angiographic vasospasm and outcome with NID. Some researchers found that NID can activate TrkB neurotrophic factor receptors to induce neuron proliferation and neuroprotective signal transduction events in the hippocampus and prefrontal cortex of mice [72], which may be illustrative. Several studies also speculated that this protective mechanism is related to the reduction of microthrombosis, inhibition of diffuse ischemia and spreading depolarizations (SD), and increase of fibrinolytic activity [73–75]. But it is clear in RCTs that it is exactly through these mechanisms instead of vasodilation that the prognosis of patients might be improved. Our study showed consistency with above results. NID did improve the prognosis of patients while it is not statistically significant for the other simple vasodilators (FSD, NCD, etc.) to be more likely to improve the prognosis, although they have reduced the frequency of cerebral vasospasm in their respective studies. It is undeniable for NID to be of prominent position clinically as the most widely used drug in aSAH patients. But unfortunately, its curative effect is mild with limited effect of improving the prognosis of postoperative patients and earlier RCTs showed that nimodipine could not improve mortality in patients with Hunt and Hess grade 4 to 5 [26]. In terms of improving patient outcomes, it is observed that nimodipine is still a promising old drug in our study.

Brain injury after aSAH is a multimodal process including early brain injury (EBI) and delayed cerebral ischemia (DCI) but mechanism of DCI is not yet fully understood [76]. The pathophysiological processes that may be involved at this stage include cerebral vasospasm (CVS), microvascular constriction, microthrombosis, diffuse cortical ischemia, and delayed apoptosis [77]. CTZ differs from conventional platelet aggregation inhibitors as apart from microthrombosis prevention, it also has a vasodilation effect by inhibiting phosphodiesterase-3 and increasing intracellular cyclic adenosine monophosphate, which mainly functions in the DCI stage of brain injury. Since pure anti-vasospasm drugs [MGS (OR = 1.43, 95%Cl 0.96,2.13), NCD (OR = 2.44, 95%Cl 0.88,6.74), CST (OR = 1.05, 95%Cl 0.60,1.85), FSD (OR = 1.74, 95%Cl 0.80,3.78)] did not show definite prognostic improvement, the efficacy of CTZ comes most likely from its anti-microthrombosis ability. Another meta-analysis [78] also showed the reduced risk of symptomatic vasospasm, cerebral infarction, and poor outcomes in the CTZ group. Currently, there are no RCTs directly comparing the efficacy of NID with CTZ on amelioration of outcome in postoperative patients with aSAH. Our study indicated that CTZ has a definite effect on the improvement of outcome which is better than NID.

ENP and ST showed a tendency to be more likely to have a poor outcome than the placebo group. For ENP, it mainly works by reducing inflammation and restoring the integrity of the blood–brain barrier [79]. We found that it could not improve patient outcomes and may aggravate the patient's condition due to its potential to increase the risk of intracranial hemorrhage. For ST, they have anti-vasospasm and anti-inflammatory effects. Vasospasm is closely related to the DCI process after aSAH, but the inflammatory mechanism runs throughout the EBI and DCI brain injury process [80–83]. Several different trials [53, 54, 58, 84] indicated that the efficacy of statins is still controversial. A meta-study including 6 RCTs and 2 prospective cohort studies also concludes that the outcome following Aneurysmal SAH was also not improved by statin treatment [85]. Given the controversy in current literature, more deterministic trials are needed to confirm the effect of ST on the prognosis of aSAH. Our study is also a expand to another large-scale meta-analysis [86], which mainly studied the clinical outcome of aSAH patients treated with CVS targeted therapy. On this basis, we included anti-inflammatory, anti-oxidant, iron chelator, anti-platelet formation, and other drug RCTs for comparative analysis. This extensive and comprehensive supplement is necessary because more and more shreds of evidence, including well-designed RCTs and summarized clinical guidelines, have shown that brain injury after aSAH is a complex pathology involving multiple factors where there is no causal connection between the simple use of drugs to reduce vasospasm and a good outcome and other drugs targeted for brain damage may also play an important role in improving the effectiveness of patients' outcome. However, current studies mainly focus on single drug application postoperatively, 13 kinds of single drug interventions in our study, only two single drug therapies showed a definite improvement in outcome and effectiveness also relatively moderate. Considering surgical intervention is effective to control the rebleeding, the application of CTZ may play the function of micro thrombosis to the maximum extent without worrying about the risk of rebleeding. Hence, it also might be worthwhile investigating whether combination treatment with other drug therapies, such as NID provides neuroprotection and CTZ to prevent microthrombosis, could be useful in the future. Interestingly, we found that recently, some scholars have designed a prospective, randomised, double-blinded, placebo-controlled trial protocol to evaluate efficacy and safety of cilostazol-nimodipine combined therapy on delayed cerebral ischaemia after aneurysmal subarachnoid haemorrhage, which may show better clinical outcomes in the future. Similarly, we suppose the potential combinability of ST. Although ST failed to show a good ability to improve clinical outcome in our study, given the important role of nerve inflammation in the process of aSAH, the relative safety of ST and the most recent AHA/ASA guidelines [70], published in 2012, noted that despite the lack of strong evidence of benefit, it makes sense to administer ST to prevent vasospasm in patients after aSAH, we believe that ST still has great potential, and may be used in combination with other drugs to produce curative effect in terms ofimproving the prognosis of patients with significant synergistic effect. In addition, another recent retrospective analysis [87] found a 24.36% improvement in cerebrovascular diameter in patients treated with multiple vasodilators compared with those treated with a single agent (P < 0.0001). Not only did it resolve cerebral vasospasm more effectively, but patients treated with multiple vasodilators also showed better improvement in the functional outcome at discharge (OR = 0.15, 95%CI 0.04–0.55; *p* = 0.004) and 90-day follow-up (OR = 0.20, 95% CI 0.05–0.77; *p* = 0.019).Studies have shown the potential benefits of multi-drug treatment strategies, but such combinations also face questions inevitably about the safety of the combined drugs and the choice of the best dose for different drugs in the multi-drug regimen, which require further study to remaining correspondingly secure in the combined application.

At present, no drugs other than NID and CTZ have a definite effect on improvement of prognosis. Nevertheless, it cannot be denied that these drugs still potentially play an improving role in the course of the disease, among which CST acting through the inhibition of endothelin A receptor antagonist, exert an effect on the reduction of angiographic vasospasm without a significant effect on the outcome, its hypotension, and pulmonary complications associated with the drug use could have counteracted the beneficial effects of the drug. Considering the lack of enough safety and efficacy, routine infusion or a combination of CST is not recommended. but in patients at risk of moderate-severe angiographic vasospasm, an appropriate amount of application may be pertinent to reduce the risk of delayed ischemic neurologic deficit (DIND) requiring rescue therapy [88]. NCD is not recommended to improve patient prognosis as it is not as effective as NID, but it can be used for patients with blood pressure management. FSD is widely used only in Japan. Its efficacy is controversial with corresponding potential in improving the prognosis of patients, and its further evaluation by RCT on a larger scale is still needed. With hemorrhage risk, routine infusion of high-dose ENP is not recommended. As glutamic acid could cause pathological SD [89], MGS provides potential neuroprotection [90, 91] by blocking the release of glutamate, so we recommend to maintain a normal dynamic balance of serum magnesium in aSAH patients, but routine infusion of magnesium above the normal level is not needed. There is no standard use for other drugs such as ω-3FA and Tirilazad, and more research is needed to evaluate them.

### Strengths and limitations

The outstanding advantage of our study is to utilize the superiority of network meta-analysis structure, comprehensively evaluates the efficacy of drugs involving various mechanisms in improving outcome, complements the lack of mutual comparison among several drugs, and ranks the efficacy of 13 drug interventions for the first time. In light of this, we believe our research has enough innovation. Secondly, it is observed that most single drug treatments have no definite efficacy, and the single drug application efficacy of NID and CTZ is relatively mild. It is put forward the idea that future studies therefore should focus on the application of combination drugs and the ranking results of efficacy in our study can provide a reference for the selection of priority combination drugs in future clinical application, which has corresponding important clinical significance.

The limitations of our study also need to be acknowledged, and several limitations may have influenced our results. First, there was significant heterogeneity in the included studies. Due to the different severity of disease in some of the included patients, for example, 14 RCTs excluded patients with Hunt-Hess scale or WFNS grade 5, while 2 RCTS excluded patients with Hunt/Hess grade or WFNS 1–2, their prognosis could differ greatly by the severity itself, so the efficacy of some intervention measures may be misestimated. Moreover, the follow-up of outcome indicators varies from 14 days to 1 year, which may give false credibility to the prognosis assessment of patients. In view of this, people may question the original intention of our NMA. We tried to analyze the results by including RCTs with complete primary outcomes measurement, which allowed us to overcome this shortcoming by using a homogeneous end point that was easy to assess. Second, we also acknowledge that some publications may have been left out, since we only include publications in English. This can lead to language bias because studies with statistically significant results are more likely to be published in English [92]. third, due to various reasons, there are not enough RCTs for drug intervention of EPO, RT-PA, ENP, MPN and other parts, so the evidence based on its efficacy is limited, which makes it more difficult for our NMA to draw a summary conclusion.

## Conclusion

In summary, our NMA showed that both CTZ and NID had definite efficacy in improving the prognosis of patients, while ENP and ST-based postoperative treatment of aSAH were the least effective interventions. Our study may provide strong evidence that CTZ is the best intervention for improving the prognosis of patients with aSAH in this particular population, and provide implications for future studies, which is that the combination of two or more drugs with relative safety and potential benefits (such as CTZ combined with NID) may improve the clinical outcome of patients more effectively.

## Supplementary Information

**Additional file 1: Figure 1.**

**Additional file 2: Figure 2.**

**Additional file 3: Figure 3.**

**Additional file 4.** Supplement.

**Additional file 5.** Supplement.

**Additional file 6.** Supplement.

**Additional file 7.** Supplement.

**Additional file 8.** Supplement.

## Data Availability

All data generated or analysed during this study are included in this published article.

## Abbreviations

aSAH: Aneurysmal Subarachnoid Hemorrhage
CI: Confidence Interval
GOS: Glasgow Outcome Scale
GOSE: Extended Glasgow Outcome Scale
mRS: Modified Rankin Scale
NMA: Network Meta-analysis
OR: Odds Ratio
RCTs: Randomized Controlled Trials
SUCRA: Surface Under the Cumulative Ranking curve
WFNS: World Federation of Neurological Surgeons grade
MGS: Magnesium
NID: Nimodipine
ST: Statins
CTZ: Cilostazol
CST: Clazosentan
TZD: Tirilazad
FSD: Fasudil
EPO: Erythropoietin
NCD: Nicardipine
ω-3FA: Omega-3 fatty acid
ENP: Enoxaparin
rt-PA: Recombinant Human Tissue-type plasminogen activator
MPN: Methylprednisolone
EBI: Early brain injury
DCI: Delayed cerebral ischemia
CVS: Cerebral vasospasm
