# Supplementary material for: Effectiveness comparisons of drug therapies for postoperative aneurysmal subarachnoid hemorrhage patients: network meta‑analysis and systematic review
Source: BMC Neurol. 2021 Jul 27;21:294. doi: 10.1186/s12883-021-02303-8 (PMC8314452; doi:10.1186/s12883-021-02303-8)
Supplement: Supplementary file 3 — Additional file 3: Figure 3. [file 12883_2021_2303_MOESM3_ESM.pdf]

Effectiveness Comparisons of Drug Therapies for Postoperative Aneurysmal Subarachnoid Hemorrhage Patients: Network Meta-analysis and systematic review

Wanli Yu[1#], MM, Yizhou Huang[2#], MM, Xiaolin Zhang[1], MM, Huirong Luo[3], Weifu Chen[1], MD, Yongxiang Jiang[1*], MD, Yuan Cheng[1*], MD

[1] Department of Neurosurgery, The Second Affiliated Hospital, Chongqing Medical University, Chongqing, China

[2] Department of Endocrinology, The Second Affiliated Hospital, Chongqing Medical University, Chongqing, China

[3] Department of Psychiatry, The First Affiliated Hospital, Chongqing Medical University, Chongqing, China

[#] Wanli Yu and Yizhou Huang contributed equally to this project.

*** Correspondence:**

Yuan Cheng, Department of Neurosurgery, The Second Affiliated Hospital, Chongqing Medical University; Yongxiang Jiang, Department of Neurosurgery, The Second Affiliated Hospital, Chongqing Medical University;

E-mail address: chengyuan@hospital.cqmu.edu.cn and doctorjiang2003@163.com

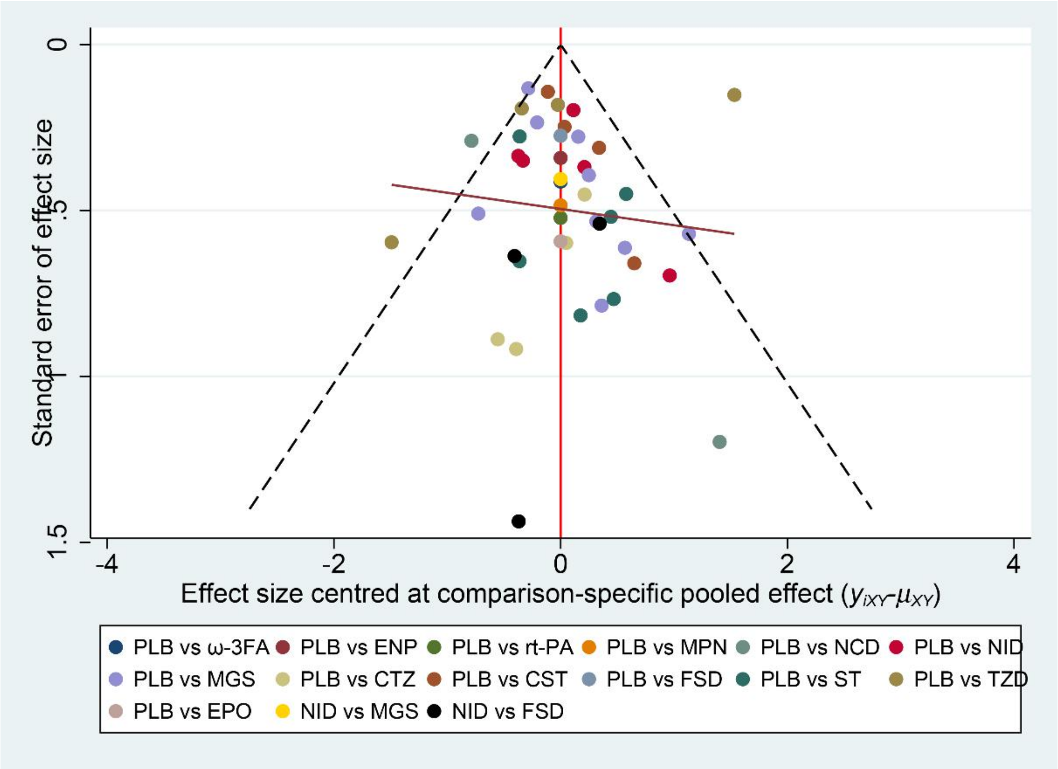

Supplement Figure.3 legend funnel plot. Red line suggests null hypothesis that study-specific effect sizes do not differ from respective comparison-specific pooled effect estimates. Different colors represent different comparisons.
