# Supplementary material for: Effectiveness comparisons of drug therapies for postoperative aneurysmal subarachnoid hemorrhage patients: network meta‑analysis and systematic review
Source: BMC Neurol. 2021 Jul 27;21:294. doi: 10.1186/s12883-021-02303-8 (PMC8314452; doi:10.1186/s12883-021-02303-8)
Supplement: Supplementary file 5 — Additional file 5. Supplement. [file 12883_2021_2303_MOESM5_ESM.pdf]

Effectiveness Comparisons of Drug Therapies for Postoperative Aneurysmal Subarachnoid Hemorrhage Patients: Network Meta-analysis and systematic review

Wanli Yu[1#], MM, Yizhou Huang[2#], MM, Xiaolin Zhang[1], MM, Huirong Luo[3], Weifu Chen[1], MD, Yongxiang Jiang[1*], MD, Yuan Cheng[1*], MD

[1] Department of Neurosurgery, The Second Affiliated Hospital, Chongqing Medical University, Chongqing, China

[2] Department of Endocrinology, The Second Affiliated Hospital, Chongqing Medical University, Chongqing, China

[3] Department of Psychiatry, The First Affiliated Hospital, Chongqing Medical University, Chongqing, China

[#] Wanli Yu and Yizhou Huang contributed equally to this project.

*** Correspondence:**

Yuan Cheng, Department of Neurosurgery, The Second Affiliated Hospital, Chongqing Medical University; Yongxiang Jiang, Department of Neurosurgery, The Second Affiliated Hospital, Chongqing Medical University;

E-mail address: chengyuan@hospital.cqmu.edu.cn and doctorjiang2003@163.com

```
# Binomial likelihood, logit link

# Random effects model for multi-arm trials

model{ # *** PROGRAM STARTS

for(i in 1:ns){ # LOOP THROUGH STUDIES

w[i,1] <- 0 # adjustment for multi-arm trials is zero for control arm

delta[i,1] <- 0 # treatment effect is zero for control arm

mu[i] ~ dnorm(0,.0001) # vague priors for all trial baselines

for (k in 1:na[i]) { # LOOP THROUGH ARMS

r[i,k] ~ dbin(p[i,k],n[i,k]) # binomial likelihood

logit(p[i,k]) <- mu[i] + delta[i,k] # model for linear predictor

rhat[i,k] <- p[i,k] * n[i,k] # expected value of the numerators

dev[i,k] <- 2 * (r[i,k] * (log(r[i,k])-log(rhat[i,k])) #Deviance contribution

+ (n[i,k]-r[i,k]) * (log(n[i,k]-r[i,k]) - log(n[i,k]-rhat[i,k])))

}

resdev[i] <- sum(dev[i,1:na[i]]) # summed residual deviance contribution for this trial

for (k in 2:na[i]) { # LOOP THROUGH ARMS

delta[i,k] ~ dnorm(md[i,k],taud[i,k]) # trial-specific LOR distributions

md[i,k] <- d[t[i,k]] - d[t[i,1]] + sw[i,k] # mean of LOR distributions (with multi-arm trial correction)

taud[i,k] <- tau *2*(k-1)/k # precision of LOR distributions (with multi-arm trial correction)

w[i,k] <- (delta[i,k] - d[t[i,k]] + d[t[i,1]]) # adjustment for multi-arm RCTs

sw[i,k] <- sum(w[i,1:k-1])/(k-1) # cumulative adjustment for multi-arm trials

}

}

totresdev <- sum(resdev[]) #Total Residual Deviance

d[1] <- 0 # treatment effect is zero for reference treatment

for (k in 2:nt){ d[k] ~ dnorm(0,.0001) } # vague priors for treatment effects

sd ~ dunif(0,5) # vague prior for between-trial SD.

tau <- pow(sd,-2) # between-trial precision = (1/between-trial variance)


# pairwise ORs and LORs for all possible pair-wise comparisons, if nt>2
```

```
for (c in 1:(nt-1)) {
for (k in (c+1):nt) {
or[c,k] <- exp(d[k] - d[c])
lor[c,k] <- (d[k]-d[c])
}
}
# Ranking and probabilities for treatment
for(k in 1:nt) {
order[k]<- nt+1-rank(d[],k)    #events are good
most.effective[k]<-equals(order[k],1)
for(j in 1:nt) {
effectiveness[k,j]<- equals(order[k],j)
cumeffectiveness[k,j]<- sum(effectiveness[k,1:j])
}
}

#SUCRA
for(k in 1:nt) {
SUCRA[k]<- sum(cumeffectiveness[k,1:(nt-1)]) /(nt-1)
}
}

list(ns=44, nt=14)                                    # tolerability
t[,1] t[,2] r[,1] n[,1] r[,2] n[,2] na[]
1 2 28 82 28 72 2
2 3 31 51 34 53 2
1 2 185 276 223 278 2
1 2 26 54 49 73 2
2 6 48 60 49 55 2
1 2 26 39 28 31 2
```

1 3 27 53 34 54 2

1 3 10 22 15 23 2

1 3 13 31 20 27 2

1 3 83 127 90 122 2

1 3 34 43 28 40 2

1 3 446 597 448 606 2

1 3 16 30 20 30 2

1 3 52 158 57 169 2

1 3 9 15 11 15 2

1 4 61 74 70 74 2

1 4 51 55 52 54 2

1 4 24 51 39 49 2

1 4 20 24 24 26 2

1 5 66 96 79 107 2

1 5 287 383 542 764 2

1 5 131 172 136 181 2

1 5 48 55 48 52 2

1 6 95 136 98 131 2

2 6 28 34 27 33 2

2 6 31 32 31 32 2

1 7 19 40 23 40 2

1 7 4 19 519 2

1 7 44 54 46 54 2

1 7 12 16 12 16 2

1 7 10 20 7 19 2

1 7 222 247 192 229 2

1 14 421 455 427 447 2

1 14 5 12 11 12 2

1 8 38 42 43 61 2

1 8 183 256 167 251 2

1 8 212 299 218 300 2

1 8 125 414 280 405 2

1 9 20 30 17 24 2

1 10 20 45 36 55 2

1 2 86 109 86 104 2

1 11 63 85 59 85 2

1 12 14 30 18 30 2

1 13 31 46 40 49 2

END

#chain 1

list(d=c(NA,0,0,0,0,0,0,0,0,0,0,0,0,0), sd=1,

mu=c(0,0,0,0,0,0,0,0,0,0,0,0,0,0,0,0,0,0,0,0,0,0,0,0,0,0,0,0,0,0,0,0,0,0,0,0,0,0,0,0,0,0,0,0))

#chain 2

list(d=c(NA,-1,-1,-1,-1,-1,-1,-1,-1,-1,-1,-1,-1,-1), sd=2,

mu=c(-3,-3,-3,-3,-3,-3,-3,-3,-3,-3,-3,-3,-3,-3,-3,-3,-3,-3,-3,-3,-3,-3,-3,-3,-3,-3,-3,-3,-3,-3,-3,-3,-3,-3,-3,

-3,-3,-3,-3,-3,-3,-3,-3,-3))

#chain 3

list(d=c(NA,2,2,2,2,2,2,2,2,2,2,2,2,2), sd=4,

mu=c(5,3,2,4,-5,-4,-1,5,5,-4,4,-2,-6,2,4,1,7,5,2,-1,-5,-3,-1,-7,-7,-7,-2,0,7,7,5,5,9,8,7,5,6,7,1,2,0,-1,5,

-5))
