# Supplementary material for: Effectiveness comparisons of drug therapies for postoperative aneurysmal subarachnoid hemorrhage patients: network meta‑analysis and systematic review
Source: BMC Neurol. 2021 Jul 27;21:294. doi: 10.1186/s12883-021-02303-8 (PMC8314452; doi:10.1186/s12883-021-02303-8)
Supplement: Supplementary file 7 — Additional file 7. Supplement. [file 12883_2021_2303_MOESM7_ESM.pdf]

Effectiveness Comparisons of Drug Therapies for Postoperative Aneurysmal Subarachnoid Hemorrhage Patients: Network Meta-analysis and systematic review

Wanli Yu[1#], MM, Yizhou Huang[2#], MM, Xiaolin Zhang[1], MM, Huirong Luo[3], Weifu Chen[1], MD, Yongxiang Jiang[1*], MD, Yuan Cheng[1*], MD

[1] Department of Neurosurgery, The Second Affiliated Hospital, Chongqing Medical University, Chongqing, China

[2] Department of Endocrinology, The Second Affiliated Hospital, Chongqing Medical University, Chongqing, China

[3] Department of Psychiatry, The First Affiliated Hospital, Chongqing Medical University, Chongqing, China

[#] Wanli Yu and Yizhou Huang contributed equally to this project.

*** Correspondence:**

Yuan Cheng, Department of Neurosurgery, The Second Affiliated Hospital, Chongqing Medical University; Yongxiang Jiang, Department of Neurosurgery, The Second Affiliated Hospital, Chongqing Medical University;

E-mail address: chengyuan@hospital.cqmu.edu.cn and doctorjiang2003@163.com

1.PubMed search strategies:

(("nimodipine"[Mesh]) OR (Bay e 9736[Title/Abstract])) OR (e 9736, Bay[Title/Abstract])) OR (Brainal[Title/Abstract])) OR (Calnit[Title/Abstract])) OR (Kenesil[Title/Abstract])) OR ("Magnesium Sulfate"[Mesh])) OR (Sulfate, Magnesium[Title/Abstract])) OR (Magnesium Sulfate, Heptahydrate[Title/Abstract])) OR (Heptahydrate Magnesium Sulfate[Title/Abstract])) OR ("Pravastatin"[Mesh])) OR (Eptastatin[Title/Abstract])) OR (Vasten[Title/Abstract])) OR (CS-514[Title/Abstract])) OR ("pitavastatin" [Supplementary Concept])) OR (itavastatin[Title/Abstract])) OR ((E,3R,5S)-7-(2-cyclopropyl-4-(4-fluorophenyl)quinolin-3-yl)-3,5-dihydroxyhept-6-enoic acid[Title/Abstract])) OR (P 872441[Title/Abstract])) OR (NK 104[Title/Abstract])) OR ("Simvastatin"[Mesh])) OR (Zocor[Title/Abstract])) OR (MK-733[Title/Abstract])) OR (Synvinolin[Title/Abstract])) OR ("Cilostazol"[Mesh])) OR (6-(4-(1-Cyclohexyl-1H-tetrazol-5-yl)butoxy)-3,4-dihydro-2(1H)-quinolinone[Title/Abstract])) OR (OPC 13013[Title/Abstract])) OR (Pletal[Title/Abstract])) OR ("clazosentan" [Supplementary Concept])) OR (VML-588[Title/Abstract])) OR (AXV-034343[Title/Abstract])) OR (Ro 61-1790[Title/Abstract])) OR ("tirilazad" [Supplementary Concept])) OR (U-74006[Title/Abstract])) OR (U 74006F[Title/Abstract])) OR (Freedox[Title/Abstract])) OR ("fasudil" [Supplementary Concept])) OR (1-(5-isoquinolinesulfonyl)homopiperazine[Title/Abstract])) OR (fasudil mesylate[Title/Abstract])) OR (HA-1077[Title/Abstract])) OR (AT 877[Title/Abstract])) OR ("Nicardipine"[Mesh])) OR (Cardene[Title/Abstract])) OR (Vasonase[Title/Abstract])) OR (Antagonil[Title/Abstract])) OR (Cardene SR[Title/Abstract])) OR (Dagan[Title/Abstract])) OR ("Erythropoietin"[Mesh])) OR ("Fatty Acids, Omega-3"[Mesh])) OR (Omega-3 Fatty Acid[Title/Abstract])) OR (Omega 3 Fatty Acid[Title/Abstract])) OR ("Enoxaparin"[Mesh])) OR (PK-10,169[Title/Abstract])) OR ("Tissue Plasminogen Activator"[Mesh])) OR

(Plasminogen Activator, Tissue[Title/Abstract])) OR (Tissue Activator D 44[Title/Abstract])) OR (Tisokinase[Title/Abstract])) OR (Tissue-Type Plasminogen Activator[Title/Abstract])) OR ("Methylprednisolone"[Mesh])) OR (Metipred[Title/Abstract])) OR (6-Methylprednisolone[Title/Abstract])) OR (Urbason[Title/Abstract])) OR (Medrol[Title/Abstract])) AND ((((("Subarachnoid Hemorrhage"[Mesh]) OR (SAH (Subarachnoid Hemorrhage)[Title/Abstract])) OR (Hemorrhage, Subarachnoid[Title/Abstract])) OR (Subarachnoid Hemorrhages[Title/Abstract])) OR (Subarachnoid Hemorrhage, Aneurysmal[Title/Abstract])) AND (randomizedcontrolledtrial[Filter])

2.Embase search strategies:

#1 'nimodipine'/exp OR 'magnesium sulfate'/exp OR 'pitavastatin'/exp OR 'simvastatin'/exp OR 'cilostazol'/exp OR 'clazosentan'/exp OR 'tirilazad'/exp OR 'fasudil'/exp OR 'erythropoietin'/exp OR 'omega 3 fatty acid'/exp OR 'enoxaparin'/exp OR 'tissue plasminogen activator'/exp OR 'methylprednisolone'/exp OR 'Bay e 9736':ab,ti OR 'Brainal':ab,ti OR 'Calnit':ab,ti OR 'Kenesil':ab,ti OR 'Sulfate, Magnesium':ab,ti OR 'Magnesium Sulfate, Heptahydrate':ab,ti OR 'Heptahydrate Magnesium Sulfate':ab,ti OR 'Eptastatin':ab,ti OR 'Vasten':ab,ti OR 'CS-514':ab,ti OR 'itavastatin':ab,ti OR '(E,3R,5S)-7-(2-cyclopropyl-4-(4-fluorophenyl)quinolin-3-yl)-3,5-dihydroxyhept-6-enoic acid':ab,ti OR 'P 872441':ab,ti OR 'NK 104':ab,ti OR 'Zocor':ab,ti OR 'MK-733':ab,ti OR 'Synvinolin':ab,ti OR '6-(4-(1-Cyclohexyl-1H-tetrazol-5-yl)butoxy)-3,4-dihydro-2(1H)-quinolinone':ab,ti OR 'OPC 13013':ab,ti OR 'Pletal':ab,ti OR 'VML-588':ab,ti OR 'AXV-034343':ab,ti OR 'Ro 61-1790':ab,ti OR 'U-74006':ab,ti OR 'Freedox':ab,ti OR '1-(5-isoquinolinesulfonyl)homopiperazine':ab,ti OR 'fasudil mesylate':ab,ti OR 'HA 1077':ab,ti OR 'AT 877':ab,ti OR 'Cardene':ab,ti OR 'Vasonase':ab,ti OR 'Antagonil':ab,ti OR 'Cardene SR':ab,ti OR 'Dagan':ab,ti OR 'Omega-3 Fatty Acid':ab,ti OR 'Acid, Omega-3 Fatty':ab,ti OR 'PK-10,169':ab,ti OR 'Plasminogen Activator, Tissue':ab,ti OR 'Tissue Activator D-44':ab,ti OR 'Tisokinase':ab,ti OR 'Tissue-Type Plasminogen Activator':ab,ti OR 'Metipred':ab,ti OR

'6-Methylprednisolone':ab,ti OR 'Urbason':ab,ti OR 'Medrol':ab,ti

#2 'subarachnoid hemorrhage'/exp OR 'SAH (Subarachnoid Hemorrhage)':ab,ti OR 'Hemorrhage, Subarachnoid':ab,ti OR 'Subarachnoid Hemorrhage, Aneurysmal':ab,ti

#3 'randomized controlled trial'/de

#4 #1 AND #2 AND #3

3.Cochrane search strategies:

#1 MeSH descriptor: [nimodipine] explode all trees OR MeSH descriptor: [Magnesium Sulfate] explode all trees OR MeSH descriptor: [Pravastatin] explode all trees OR MeSH descriptor: [pitavastatin] explode all trees OR MeSH descriptor: [Simvastatin] explode all trees OR MeSH descriptor: [Cilostazol] explode all trees OR MeSH descriptor: [clazosentan] explode all trees OR MeSH descriptor: [tirilazad] explode all trees OR MeSH descriptor: [fasudil] explode all trees OR MeSH descriptor: [Nicardipine] explode all trees OR MeSH descriptor: [Erythropoietin] explode all trees OR MeSH descriptor: [Fatty Acids, Omega-3] explode all trees OR MeSH descriptor: [Enoxaparin] explode all trees OR MeSH descriptor: [Tissue Plasminogen Activator] explode all trees OR MeSH descriptor: [Methylprednisolone] explode all trees

#2 Bay e 9736 OR e 9736, Bay OR Brainal OR Calnit OR Kenesil OR Sulfate, Magnesium OR Magnesium Sulfate, Heptahydrate OR Eptastatin OR Vasten OR CS-514 OR itavastatin OR P 872441 OR NK 104 OR Zocor OR MK-733 OR Synvinolin OR OPC 13013 OR Pletal ORVML-588 OR AXV-034343 OR Ro 611790 OR U74006 OR U74006F OR Freedox OR fasudil mesylate OR HA 1077 OR AT 877 OR Cardene OR Vasonase OR Antagonil OR Cardene SR OR Dagan OR Omega-3 Fatty Acid OR Omega 3 Fatty Acid OR Enoxaparine OR PK-10,169 OR Plasminogen Activator, Tissue OR Tissue Activator D-44 OR Tisokinase OR Metipred OR 6 Methylprednisolone OR Urbason OR Medrol

#3 MeSH descriptor: [Subarachnoid Hemorrhage] explode all trees

#4 SAH (Subarachnoid Hemorrhage) OR Hemorrhage, Subarachnoid OR Subarachnoid Hemorrhage, Aneurysmal OR Subarachnoid Hemorrhages

#5 #1 OR #2

#6 #3 OR #4

#7 #5 AND #6
