# Supplementary material for: Effectiveness comparisons of drug therapies for postoperative aneurysmal subarachnoid hemorrhage patients: network meta‑analysis and systematic review
Source: BMC Neurol. 2021 Jul 27;21:294. doi: 10.1186/s12883-021-02303-8 (PMC8314452; doi:10.1186/s12883-021-02303-8)
Supplement: Supplementary file 8 — Additional file 8. Supplement. [file 12883_2021_2303_MOESM8_ESM.pdf]

Effectiveness Comparisons of Drug Therapies for Postoperative Aneurysmal Subarachnoid Hemorrhage Patients: Network Meta-analysis and systematic review

Wanli Yu[1#], MM, Yizhou Huang[2#], MM, Xiaolin Zhang[1], MM, Huirong Luo[3], Weifu Chen[1], MD, Yongxiang Jiang[1*], MD, Yuan Cheng[1*], MD

[1] Department of Neurosurgery, The Second Affiliated Hospital, Chongqing Medical University, Chongqing, China

[2] Department of Endocrinology, The Second Affiliated Hospital, Chongqing Medical University, Chongqing, China

[3] Department of Psychiatry, The First Affiliated Hospital, Chongqing Medical University, Chongqing, China

[#] Wanli Yu and Yizhou Huang contributed equally to this project.

*** Correspondence:**

Yuan Cheng, Department of Neurosurgery, The Second Affiliated Hospital, Chongqing Medical University; Yongxiang Jiang, Department of Neurosurgery, The Second Affiliated Hospital, Chongqing Medical University;

E-mail address: chengyuan@hospital.cqmu.edu.cn and doctorjiang2003@163.com

1. Petruk KC, West M, Mohr G, et al. Nimodipine treatment in poor-grade aneurysm patients. Results of a multicenter double-blind placebo-controlled trial. *Journal of neurosurgery.* 1988;68(4):505-517.
2. Schmid-Elsaesser R, Kunz M, Zausinger S, Prueckner S, Briegel J, Steiger HJ. Intravenous magnesium versus nimodipine in the treatment of patients with aneurysmal subarachnoid hemorrhage: a randomized study. *Neurosurgery.* 2006;58(6):1054-1065; discussion 1054-1065.
3. Pickard JD, Murray GD, Illingworth R, et al. Effect of oral nimodipine on cerebral infarction and outcome after subarachnoid haemorrhage: British aneurysm nimodipine trial. *BMJ (Clinical research ed).* 1989;298(6674):636-642.
4. Jan M, Buchheit F, Tremoulet M. Therapeutic trial of intravenous nimodipine in patients with established cerebral vasospasm after rupture of intracranial aneurysms. *Neurosurgery.* 1988;23(2):154-157.
5. Ohman J, Servo A, Heiskanen O. Long-term effects of nimodipine on cerebral infarcts and outcome after aneurysmal subarachnoid hemorrhage and surgery. *Journal of neurosurgery.* 1991;74(1):8-13.
6. Zhao J, Zhou D, Guo J, et al. Efficacy and safety of fasudil in patients with subarachnoid hemorrhage: final results of a randomized trial of fasudil versus nimodipine. *Neurologia medico-chirurgica.* 2011;51(10):679-683.
7. Philippon J, Grob R, Dagreou F, Guggiari M, Rivierez M, Viars P. Prevention of vasospasm in subarachnoid haemorrhage. A controlled study with nimodipine. *Acta neurochirurgica.* 1986;82(3-4):110-114.
8. Westermaier T, Stetter C, Vince GH, et al. Prophylactic intravenous magnesium sulfate for treatment of aneurysmal subarachnoid hemorrhage: a randomized, placebo-controlled, clinical study. *Critical care medicine.* 2010;38(5):1284-1290.
9. Boet R, Chan MT, Poon WS, Wong GK, Wong HT, Gin T. Intravenous magnesium sulfate to improve outcome after aneurysmal subarachnoid hemorrhage: interim report from a pilot study. *Acta neurochirurgica Supplement.* 2005;95:263-264.
10. Muroi C, Terzic A, Fortunati M, Yonekawa Y, Keller E. Magnesium sulfate in the management of patients with aneurysmal subarachnoid hemorrhage: a randomized, placebo-controlled, dose-adapted trial. *Surgical neurology.* 2008;69(1):33-39; discussion 39.
11. van den Bergh WM, Algra A, van Kooten F, et al. Magnesium sulfate in aneurysmal subarachnoid hemorrhage: a randomized controlled trial. *Stroke.* 2005;36(5):1011-1015.
12. Akdemir H, Kulakszoğlu EO, Tucer B, Menkü A, Postalc L, Günald Ö. Magnesium sulfate therapy for cerebral vasospasm after aneurysmal subarachnoid hemorrhage. *Neurosurgery Quarterly.* 2009;19(1):35-39.
13. Dorhout Mees SM, Algra A, Vandertop WP, et al. Magnesium for aneurysmal subarachnoid haemorrhage (MASH-2): a randomised placebo-controlled trial. *Lancet (London, England).* 2012;380(9836):44-49.
14. Wong GK, Chan MT, Boet R, Poon WS, Gin T. Intravenous magnesium sulfate after aneurysmal subarachnoid hemorrhage: a prospective randomized pilot study. *J Neurosurg Anesthesiol.* 2006;18(2):142-148.
15. Wong GK, Poon WS, Chan MT, et al. Intravenous magnesium sulphate for aneurysmal subarachnoid hemorrhage (IMASH): a randomized, double-blinded, placebo-controlled,

multicenter phase III trial. *Stroke.* 2010;41(5):921-926.
16. Hassan T, Nassar M, Elhadi SM, Radi WK. Effect of magnesium sulfate therapy on patients with aneurysmal subarachnoid hemorrhage using serum S100B protein as a prognostic marker. *Neurosurg Rev.* 2012;35(3):421-427; discussion 427.
17. Matsuda N, Naraoka M, Ohkuma H, et al. Effect of Cilostazol on Cerebral Vasospasm and Outcome in Patients with Aneurysmal Subarachnoid Hemorrhage: A Randomized, Double-Blind, Placebo-Controlled Trial. *Cerebrovascular diseases (Basel, Switzerland).* 2016;42(1-2):97-105.
18. Senbokuya N, Kinouchi H, Kanemaru K, et al. Effects of cilostazol on cerebral vasospasm after aneurysmal subarachnoid hemorrhage: a multicenter prospective, randomized, open-label blinded end point trial. *Journal of neurosurgery.* 2013;118(1):121-130.
19. Suzuki S, Sayama T, Nakamura T, et al. Cilostazol improves outcome after subarachnoid hemorrhage: a preliminary report. *Cerebrovascular diseases (Basel, Switzerland).* 2011;32(1):89-93.
20. Yoshimoto T, Shirasaka T, Fujimoto S, et al. Cilostazol may prevent cerebral vasospasm following subarachnoid hemorrhage. *Neurologia medico-chirurgica.* 2009;49(6):235-240; discussion 240-231.
21. Macdonald RL, Kassell NF, Mayer S, et al. Clazosentan to overcome neurological ischemia and infarction occurring after subarachnoid hemorrhage (CONSCIOUS-1): randomized, double-blind, placebo-controlled phase 2 dose-finding trial. *Stroke.* 2008;39(11):3015-3021.
22. Macdonald RL, Higashida RT, Keller E, et al. Clazosentan, an endothelin receptor antagonist, in patients with aneurysmal subarachnoid haemorrhage undergoing surgical clipping: a randomised, double-blind, placebo-controlled phase 3 trial (CONSCIOUS-2). *The Lancet Neurology.* 2011;10(7):618-625.
23. Macdonald RL, Higashida RT, Keller E, et al. Randomized trial of clazosentan in patients with aneurysmal subarachnoid hemorrhage undergoing endovascular coiling. *Stroke.* 2012;43(6):1463-1469.
24. Fujimura M, Joo JY, Kim JS, Hatta M, Yokoyama Y, Tominaga T. Preventive Effect of Clazosentan against Cerebral Vasospasm after Clipping Surgery for Aneurysmal Subarachnoid Hemorrhage in Japanese and Korean Patients. *Cerebrovascular diseases (Basel, Switzerland).* 2017;44(1-2):59-67.
25. Shibuya M, Suzuki Y, Sugita K, et al. Effect of AT877 on cerebral vasospasm after aneurysmal subarachnoid hemorrhage. Results of a prospective placebo-controlled double-blind trial. *Journal of neurosurgery.* 1992;76(4):571-577.
26. Zhao J, Zhou D, Guo J, et al. Effect of fasudil hydrochloride, a protein kinase inhibitor, on cerebral vasospasm and delayed cerebral ischemic symptoms after aneurysmal subarachnoid hemorrhage. *Neurologia medico-chirurgica.* 2006;46(9):421-428.
27. Jingjian. M, Shuyuan. Y, Guoliang. H. Effect of fasudil hydrochloride on cerebral vasospasm following aneurysmal subarachnoid hemorrhage in phase Ⅱ clinical trial. *Journal of chinese clinical medicine.* 2009;4(2).
28. Tseng MY, Czosnyka M, Richards H, Pickard JD, Kirkpatrick PJ. Effects of acute treatment with pravastatin on cerebral vasospasm, autoregulation, and delayed ischemic deficits after aneurysmal subarachnoid hemorrhage: a phase II randomized placebo-controlled trial. *Stroke.* 2005;36(8):1627-1632.

29. Garg K, Sinha S, Kale SS, et al. Role of simvastatin in prevention of vasospasm and improving functional outcome after aneurysmal sub-arachnoid hemorrhage: a prospective, randomized, double-blind, placebo-controlled pilot trial. *British journal of neurosurgery.* 2013;27(2):181-186.
30. Naraoka M, Matsuda N, Shimamura N, et al. Long-acting statin for aneurysmal subarachnoid hemorrhage: A randomized, double-blind, placebo-controlled trial. *Journal of cerebral blood flow and metabolism : official journal of the International Society of Cerebral Blood Flow and Metabolism.* 2018;38(7):1190-1198.
31. Vergouwen MD, Meijers JC, Geskus RB, et al. Biologic effects of simvastatin in patients with aneurysmal subarachnoid hemorrhage: a double-blind, placebo-controlled randomized trial. *Journal of cerebral blood flow and metabolism : official journal of the International Society of Cerebral Blood Flow and Metabolism.* 2009;29(8):1444-1453.
32. Chou SH, Smith EE, Badjatia N, et al. A randomized, double-blind, placebo-controlled pilot study of simvastatin in aneurysmal subarachnoid hemorrhage. *Stroke.* 2008;39(10):2891-2893.
33. Kirkpatrick PJ, Turner CL, Smith C, Hutchinson PJ, Murray GD. Simvastatin in aneurysmal subarachnoid haemorrhage (STASH): a multicentre randomised phase 3 trial. *The Lancet Neurology.* 2014;13(7):666-675.
34. Haley EC, Jr., Kassell NF, Torner JC. A randomized controlled trial of high-dose intravenous nicardipine in aneurysmal subarachnoid hemorrhage. A report of the Cooperative Aneurysm Study. *Journal of neurosurgery.* 1993;78(4):537-547.
35. Barth M, Capelle HH, Weidauer S, et al. Effect of nicardipine prolonged-release implants on cerebral vasospasm and clinical outcome after severe aneurysmal subarachnoid hemorrhage: a prospective, randomized, double-blind phase IIa study. *Stroke.* 2007;38(2):330-336.
36. Haley EC, Jr., Kassell NF, Alves WM, Weir BK, Hansen CA. Phase II trial of tirilazad in aneurysmal subarachnoid hemorrhage. A report of the Cooperative Aneurysm Study. *Journal of neurosurgery.* 1995;82(5):786-790.
37. Kassell NF, Haley EC, Jr., Apperson-Hansen C, Alves WM. Randomized, double-blind, vehicle-controlled trial of tirilazad mesylate in patients with aneurysmal subarachnoid hemorrhage: a cooperative study in Europe, Australia, and New Zealand. *Journal of neurosurgery.* 1996;84(2):221-228.
38. Haley EC, Jr., Kassell NF, Apperson-Hansen C, Maile MH, Alves WM. A randomized, double-blind, vehicle-controlled trial of tirilazad mesylate in patients with aneurysmal subarachnoid hemorrhage: a cooperative study in North America. *Journal of neurosurgery.* 1997;86(3):467-474.
39. Lanzino G, Kassell NF, Dorsch NW, et al. Double-blind, randomized, vehicle-controlled study of high-dose tirilazad mesylate in women with aneurysmal subarachnoid hemorrhage. Part I. A cooperative study in Europe, Australia, New Zealand, and South Africa. *Journal of neurosurgery.* 1999;90(6):1011-1017.
40. Springborg JB, Møller C, Gideon P, Jørgensen OS, Juhler M, Olsen NV. Erythropoietin in patients with aneurysmal subarachnoid haemorrhage: a double blind randomised clinical trial. *Acta neurochirurgica.* 2007;149(11):1089-1101; discussion 1101.
41. Nakagawa I, Yokoyama S, Omoto K, et al. ω-3 Fatty Acids Ethyl Esters Suppress Cerebral Vasospasm and Improve Clinical Outcome Following Aneurysmal Subarachnoid Hemorrhage.

World neurosurgery. 2017;99:457-464.

42. Siironen J, Juvela S, Varis J, et al. No effect of enoxaparin on outcome of aneurysmal subarachnoid hemorrhage: a randomized, double-blind, placebo-controlled clinical trial. *Journal of neurosurgery.* 2003;99(6):953-959.

43. Gomis P, Graftieaux JP, Sercombe R, Hettler D, Scherpereel B, Rousseaux P. Randomized, double-blind, placebo-controlled, pilot trial of high-dose methylprednisolone in aneurysmal subarachnoid hemorrhage. *Journal of neurosurgery.* 2010;112(3):681-688.

44. Etminan N, Beseoglu K, Eicker SO, Turowski B, Steiger HJ, Hänggi D. Prospective, randomized, open-label phase ii trial on concomitant intraventricular fibrinolysis and low-frequency rotation after severe subarachnoid hemorrhage. *Stroke.* 2013;44(8):2162-2168.
